# Coming Developments in Port Sanitary Administration

**Published:** 1920-09-25

**Authors:** 


### A NATIONAL LINE OF DEFENCE.

### Coming Developments in Port Sanitary Administration.

At the close of the European phases of the war there arose opportunity, and, indeed, urgent necessity for discussion as to the ways in which disease could be systematically prevented from entering this country from abroad, as to how far our previous attempts had been successful, and as to what administrative changes were then essential to keep our lines of defence in a state of perfection commensurate with the greatly changed conditions of world travel and transport.

### War-time Illustrations.

For some time it had been realised that these matters were gaining not only a national but an international importance, and numerous war-time illustrations have fortunately provided a timely stimulus towards producing the actual and official measures for a better scientific control of the port sanitary areas. It is unnecessary here to recount the history of this coastal defence against disease, for its triumphs, and many, indeed, of its past failures, have come prominently to the public notice within recent times; but in justice to those in the public health service who have been responsible for its earlier development, it must be pointed out that efficiency in port sanitary work is by no means the newly discovered ideal that some organs of the daily press would lead us to believe. There have been years of strenuous endeavour on the part of a few men of both scientific and administrative ability, and their achievements have provided an invaluable basis upon which to build the newest and, we hope, more perfect schemes. The real source from which to-day's reforms will derive their value lies in the opportunity to correlate a host of vital experiences and to weld them by a central administration such as the Ministry of Health can now provide.

### The Situation To-day.

The situation as it stands at the moment may come as a surprise to the general reader because, although, in the case of plague, cholera, and yellow fever, Port Sanitary Authorities are officially required to administer special regulations made under the Public Health Acts by the Local Government Board in accordance with obligations imposed by the International Sanitary Convention of 1903, yet there are a number of other acutely infectious diseases for defence against which no definite and official regulations have been made. Small-pox and the much-talked-of typhus fever itself are found in this category, although it will, in view of the amount of investigational work and prominence which has been given to the subject in the last few years, be readily understood that these infectious conditions are, in local practice, dealt with as stringently as possible through the existing powers. Nevertheless, as the Ministry's annual report remarks, in view of the resumption of our great and increasing trade and passenger traffic with the Continent, and especially with those countries in which small-pox, typhus fever, and other acutely infectious diseases are seriously prevalent, great extension and improvement of the existing defence system is essential if we are still to ward off the danger of serious disease introduction to the country.

### The New Regulations.

It is highly satisfactory, therefore, that regulations are prepared, and about to be issued, conferring many additional powers and imposing further duties on the Sanitary Authorities and their officers in regard to infectious disease in ships. The national value of this enlightened and centralised control will undoubtedly be enormous, and the Ministry have already received the sanction of the Treasury to a grant in aid equal to 50 per cent. of the approved net expenditure of the Port and Riparian Sanitary Authorities, this grant becoming available as from the date on which the new regulations came into operation.

One reform in these matters is particularly noteworthy. In the past, under the provisions of the Public Health Acts, the L.G.B. had constituted all the larger seaports in England and Wales port sanitary districts under the jurisdiction of Port Sanitary Authorities, and the whole of the coastline and shores of navigable rivers are included in either port or riparian districts. On March 31, 1920, there were in all sixty of these port sanitary authorities, one of which had appointed two medical officers of health, while the rest had one officer for their districts. The stated official opinion, and one, indeed, with which most will agree, is that difficulties in administration frequently arise where the M.O.H. for the borough or district adjoining the port itself does not act also as M.O.H. for the Port Sanitary Authority. This unified local control has not everywhere existed, and during the past year the Ministry has already refused, in cases where these difficulties appeared likely to be serious, to sanction the proposal by the port authorities to appoint their own separate medical officer. On March 31, 1920, there were eighteen cases where divided control existed.

On the important question, too, of proper inspection of aliens landing in this country, an attempt is being made to bring out more direct, and therefore more perfect, administration. The port authorities concerned were asked to agree to the appointment by the Minister of their local M.O.H. as Medical Inspector of Aliens also, all expenditure thus incurred, with the approval of the Ministry, to be borne by the Exchequer. As a result schemes have been elaborated for providing a far better inspection service at each of the approved aliens' ports.

Thus with satisfaction it may be seen that in every direction the sanitary work in our many great seaboard towns is likely in the immediate future to improve by that degree which has been badly needed for some years past. Exact and legal details of the new regulations would in any case be tedious to the general reader, but enough has been said to impress the recent existence of a great national necessity, and to illustrate the steps to meet it which the Health Ministry is most advisedly taking.

